# Drawing a line between histone demethylase KDM5A and KDM5B: their roles in development and tumorigenesis

**DOI:** 10.1038/s12276-022-00902-0

**Published:** 2022-12-12

**Authors:** Jung Yoo, Go Woon Kim, Yu Hyun Jeon, Ji Yoon Kim, Sang Wu Lee, So Hee Kwon

**Affiliations:** College of Pharmacy, Yonsei Institute of Pharmaceutical Sciences, Yonsei University, Incheon, 21983 Republic of Korea

**Keywords:** Methylation, Tumour biomarkers

## Abstract

Distinct epigenetic modifiers ensure coordinated control over genes that govern a myriad of cellular processes. Growing evidence shows that dynamic regulation of histone methylation is critical for almost all stages of development. Notably, the KDM5 subfamily of histone lysine-specific demethylases plays essential roles in the proper development and differentiation of tissues, and aberrant regulation of KDM5 proteins during development can lead to chronic developmental defects and even cancer. In this review, we adopt a unique perspective regarding the context-dependent roles of KDM5A and KDM5B in development and tumorigenesis. It is well known that these two proteins show a high degree of sequence homology, with overlapping functions. However, we provide deeper insights into their substrate specificity and distinctive function in gene regulation that at times divert from each other. We also highlight both the possibility of targeting KDM5A and KDM5B to improve cancer treatment and the limitations that must be overcome to increase the efficacy of current drugs.

## Epigenetics: Roles of related enzymes in development and cancer

Two enzymes that catalyze the removal of methyl groups (-CH_3_) from the histone proteins that bind to and package DNA within chromosomes play subtly different roles in controlling gene activity in normal development and in the onset of some cancers. So Hee Kwon and colleagues at Yonsei University in Incheon, South Korea, review the activities of the closely related ‘demethylase’ proteins known as KDM5A and KDM5B. They appraise mounting evidence supporting involvement of the proteins in maintaining stable healthy development of several key tissues including brain, bone, muscle and sex organs. They also discuss the significance of aberrations in the proteins’ activities for tumor development, and the possibilities of using the proteins as biomarkers for cancer. Drugs targeting KDM5A and KDM5B could be explored as a novel approach to treating some cancers.

## Introduction

Histone modifications and chromatin-modifying enzymes have emerged as indispensable regulators of gene expression. Histone proteins are subject to posttranslational modifications such as methylation, acetylation, citrullination, phosphorylation, ubiquitination, and sumoylation^1,2^. Among the numerous enzymes that participate in the dynamic control of histone modifications, lysine-specific histone methyltransferases (KMTs) add methylation marks on different proteins^3^, whereas histone lysine demethylases (KDMs) remove them^4^. Histone methylation plays key roles in transcription and genomic stability in various organisms, including humans^5,6^. Mono-, di-, or trimethylation occurs on histone lysine residues, and each mark leads to different functions depending on the position and the number^2^. As a result, deregulation of chromatin-modifying enzymes is strongly linked to the development of various physiological diseases, including cancer.

**Fig. 1:**
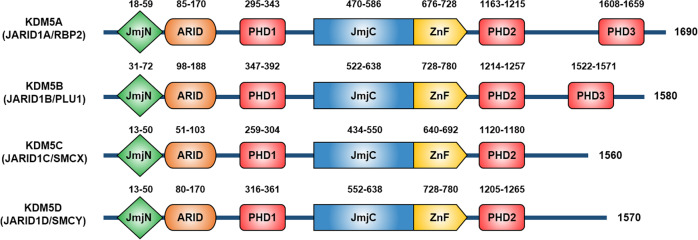
Functional domain of human KDM5 proteins. The lysine-specific histone demethylase 5 (KDM5) family is composed of 4 proteins: KDM5A, KDM5B, KDM5C, and KDM5D. Functional domain structures of KDM5 proteins show that all KDM5 proteins contain a common Jumonji-N (JmjN) domain (green), AT-rich interaction (ARID) domain (orange), plant homeodomain (PHD) 1 domain (red), Jumonji-C (JmjC) domain (blue), and zinc-finger (ZnF) domain (yellow). Differences between the four proteins arise from the presence of PHD2 and PHD3 domains. KDM5A and KDM5B have PHD2 and PHD3 domains, whereas KDM5C and KDM5D only have the PHD2 domain. The numbers indicate the location of each domain and the size of each protein in terms of amino acid residues.

Among Jumonji C domain (JMJD)-containing KDMs, the KDM5 subfamily catalyzes the removal of H3K4 di- and trimethylation (H3K4me2/3) marks, which are strongly associated with transcriptional activation^7^. The four KDM5 members are KDM5A (JARID1A/RBP2), KDM5B (JARID1B/PLU1), KDM5C (JARID1C/SMCX), and KDM5D (JARID1D/SMCY) (Fig. 1). The genes encoding KDM5A and KDM5B are located on an autosome and have a third plant homeodomain domain (PHD3). In contrast, the genes encoding KDM5C and KDM5D are located on the X and Y chromosomes, respectively, sharing biological functions and containing only two PHD domains^8,9^. The functions of the individual domains of KDM5 have been described in detail elsewhere^10^.

As KDM5A and KDM5B share a common structure and sequence homology, many reviews discuss the diverse biological functions performed by these two KDM5 proteins under different physiological contexts. However, the individual roles of KDM5A and KDM5B in a similar biological context have not been addressed by reviews to date. Many epigenetic modulators within the same family share high-sequence homology, have similar structures, and target the same epigenetic modification. This characteristic is not limited to KDM5A and KDM5B. Despite their similarity, functional differences could arise from the following points. First, although both KDM5A and KDM5B target identical histone methylation marks, their target genes can vary depending on the circumstances. RNA-sequencing data for cancer cells depleted of KDM5A or KDM5B demonstrate the heterogeneity of their target genes^11,12^. Second, KDM5 family proteins mostly exert demethylase activities on many genes, but a number of studies also emphasize the functional importance of the demethylase-independent functions of KDM5 for gene regulation^13,14^. The demethylase-independent functions of KDM5A and KDM5B involve interactions with other proteins, and depending on their interacting partners, KDM5A and KDM5B may exhibit unique functions that are not fully attributable to their demethylase activities. Recognizing these differences, we aimed to focus on comprehensively refining the roles of KDM5A and KDM5B in the context of development and cancer progression.

## Epigenetic regulation of development by KDM5A and KDM5B

Various in vitro and in vivo studies have shown that KDM5A and KDM5B both play crucial roles in regulating developmental processes, including those of stem cells, germ cells, brain, muscle, bone, blood, and adipocytes. Both KDM5A and KDM5B are classified into the same JMJD subfamily and have the same domain architecture; hence, their catalytic activity in different biological functions may overlap. Although the two proteins may have overlapping functions under certain circumstances, their differences must also be considered to elucidate their roles as novel epigenetic targets for treating various diseases.

### Regulation of cell cycle genes

During differentiation and development, most stem cells exit the cell cycle at the G_1_ checkpoint and enter the G_0_ phase, or quiescent state, to serve as a reservoir for tissue renewal and repair^15^. Prolonged G_1_ phase of the cell cycle is a hallmark of differentiation; in contrast, a shortened G_1_ phase is associated with pluripotency in both mice and humans^16,17^. Several studies hypothesize that lengthening of the G_1_ phase is required for accumulation of factors necessary for differentiation to occur^15^. As a result, cells gain two new properties upon differentiation: repression of cell cycle genes for permanent cell cycle exit and activation of cell type-specific genes.

Regulation of the cell cycle by KDM5 family proteins, such as KDM5A and KDM5B, has been suggested to play cell context dependent, paradoxical roles. One study reported that KDM5A represses cell cycle genes during differentiation in mouse embryonic stem cells (mESCs) and cooperates with E2F4 to cause cell cycle gene inactivation^18^. KDM5A knockdown leads to twofold increases in H3K4 methylation of cell cycle-promoting regulators, proliferating cell nuclear antigen and nucleolar spindle-associated protein 1, thereby inactivating these genes at their promoters in differentiated histiocytic lymphoma U937 cells. In addition to regulating cell cycle regulators, KDM5A is known to preferentially bind to transcription start site (TSS) regions of the cell cycle genes *MFN2, BRD8/KIF20A, FGF4, OTX2, HOXA1, HOXA6*, and *HOXA718–21*^18–21^. Whether these genes are directly controlled by removal of H3K4 trimethylation by KDM5A needs to be further studied. Unlike the repression of cell cycle genes during differentiation, cell cycle genes are activated by KDM5A during adipogenesis^13^. KDM5A binds to the area near the TSS of cell cycle genes during adipocyte differentiation and primes the promoters for activation during early adipogenesis. These genes include cell cycle progression genes such as *cell division cycle 6* (*Cdc6*) and *Cdc20*, which are required for mitotic clonal expansion during adipogenesis. Indeed, knockdown of KDM5A, KDM5B, and KDM5C abrogates induction of these pro-proliferative cell cycle genes in response to adipogenesis. Future research should delineate the molecular mechanisms underlying the dual role of KDM5A as a gene activator and repressor^22^.

**Fig. 2:**
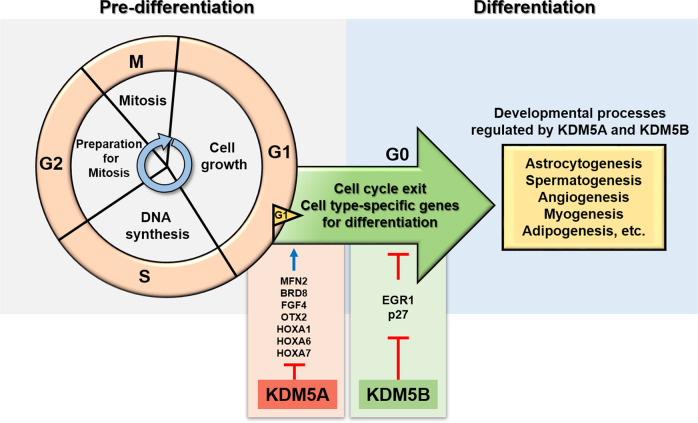
Epigenetic control of cell cycle genes by KDM5A and KDM5B during differentiation. KDM5A and KDM5B control cell cycle genes during differentiation. Under predifferentiation conditions, stem cells pass through indefinite rounds of the cell cycle. For differentiation and development to occur, cell cycle genes need to be repressed for permanent cell cycle exit, and cell type-specific genes must be expressed. A shortened G_1_ phase is associated with pluripotency in cells; a lengthened G_1_ phase marks cells for differentiation. During differentiation, KDM5A represses cell cycle genes and promotes cell cycle exit. Contrary to KDM5A, KDM5B inhibits cell cycle exit, maintaining the uncommitted state of progenitor cells by repressing expression of cell cycle inhibitors and several differentiation markers. Developmental processes that are regulated by KDM5A and KDM5B include astrocytogenesis, spermatogenesis, angiogenesis, myogenesis, and adipogenesis.

Similar to KDM5A, KDM5B plays a decisive role in the regulation of cell fate. In mESCs, KDM5B directly removes H3K4me3 from the promoters of genes involved in the cell cycle and cell lineage control. KDM5B contributes to development by maintaining an uncommitted state of progenitors^23^. For example, knockdown of KDM5B in mESCs significantly increases mRNA levels of *BMI1*, a neural cell lineage marker, *Egr1*, a cell differentiation marker, and p27, a cell cycle inhibitor. Overexpression of KDM5B decreases the expression level of these genes, maintaining the pluripotent state of undifferentiated stem cells. Constitutive expression of KDM5B in mESCs ensures that these genes are repressed to prevent improper differentiation. Although the aforementioned studies indicate that KDM5A and KDM5B both regulate cell cycle genes during development, they appear to play opposite roles in cell cycle regulation during differentiation (Fig. 2): KDM5B induces stem cell proliferation and inhibits cell cycle exit^23,24^, whereas KDM5A promotes differentiation by promoting cell cycle exit^25^ and progression^13^. For KDM5A and KDM5B, various cellular contexts are considered in cell cycle modulation during differentiation, and further studies should be conducted collectively.

### Regulation of different developmental genes

**Fig. 3:**
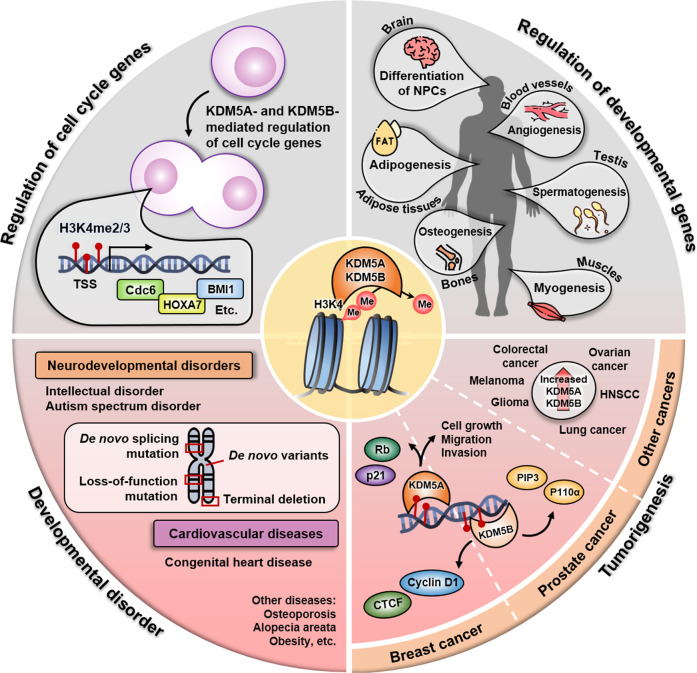
Schematic summary of the various roles KDM5A and KDM5B play in development and cancer. The KDM5A and KDM5B proteins both remove histone 3 lysine 4 trimethylation (H3K4me3) activation marks at transcription start sites (TSSs). The two proteins play various physiological roles by removing the methylation marks of different cell cycle genes and developmental genes. As a result, dysregulation of KDM5A and KDM5B leads to multiple developmental disorders, such as neurodevelopmental disorders and congenital heart diseases. Malfunction of the two proteins also promotes tumorigenesis in different cancers, including breast cancer and prostate cancer, by inducing uncontrolled cell growth, migration, and invasion. The two proteins are known to be upregulated in many other cancers aside from breast cancer and prostate cancer.

Development occurs as stem cells become committed to serving a specialized function in the body. Stem cells are well known for their pluripotency and ability to differentiate into different types of cells. The generation of specific cell types from pluripotent stem cells requires precise and timely expression of a variety of genes. For precise control of gene expression during differentiation, epigenetic regulation mechanisms play indispensable roles. Among the several proteins involved in epigenetic control, KDM5A and KDM5B remove H3K4 methylation marks on different TSSs of genes that participate in astrocytogenesis^26^, spermatogenesis^27–29^, angiogenesis^30,31^, myogenesis^32,33^, and adipogenesis^13,34^ (Fig. 3).

#### Brain development

KDM5A has previously been described to promote differentiation of cells into mESCs^18^. However, KDM5A during brain development represses neural progenitor cells (NPCs) differentiation^26^. NPCs differentiate into neurons, astrocytes, and oligodendrocytes^35^. Kong and colleagues demonstrated that KDM5A represses astrocyte differentiation in NPCs by removing H3K4 methyl marks at the TSS of *Gfap*, an astroglial gene. Knockdown of KDM5A decreases its recruitment to the *Gfap* promoter, and the protein levels of KDM5A are lower in differentiated cells than in NPCs. On the other hand, KDM5A overexpression reduces the transcriptional activity of the *Gfap* promoter. KDM5A prevents astrocyte differentiation in NPCs, but KDM5B helps to promote differentiation of NPCs in neural development^36^. H3K4 methylation marks, which are substrates of KDM5B, at the TSS of neural stem cell marker genes, such as *Nanog* and *Rnf17*, are lost during neural differentiation. Kdm5b functions to aid in neural stem cell gene silencing and helps to add silencing methylation marks such as H3K27me3. Indeed, Kdm5b-depleted ESCs retain H3K4me3 marks, and stem cell- and germ cell-specific genes are not fully silenced during neural differentiation. Incomplete silencing of lineage-inappropriate genes might hamper NPCs from further commitment toward mature neuronal cells. As seen from these studies, it is evident that KDM5A and KDM5B are essential in brain development and differentiation. Further studies are required to strongly proclaim the roles of KDM5A and KDM5B in neural stem cell differentiation.

#### Spermatogenesis

KDM5A and KDM5B play crucial roles in spermatogenesis, an ongoing differentiation process that occurs in the male testis to produce germ cells. A recent study showed that *Kdm5a* is involved in differentiation of gonocytes into spermatogonial stem cells (SSCs)^27^, which further differentiate into spermatozoa^37^. The importance of *Kdm5a* is implied by the ubiquitous expression of *Kdm5a* in the nuclei of gonocytes, spermatogonia, and spermatocytes. Overexpression of *Kdm5a* in mouse spermatogonial cells alters H3K4me3/me2 epigenetic marks on five genes related to SSC development (*Esr2, Neurog3, Pou5f1, Ret*, and *Thy)* and increases their transcription levels^27^. However, deeper studies are needed to fully understand the exact role of KDM5A in spermatogenesis.

Studies have shown that KDM5B is highly expressed in mitotic spermatogonia and the testis^28,29^. In line with the role of KDM5B in maintaining stem cell pluripotency by inhibiting termination of the cell cycle in mESCs^23,24^, KDM5B inactivation induces differentiation of spermatogonia into spermatocytes^38^. In the same sense, high levels of *KDM5B* mRNA correlate positively with increased differentiating spermatocytes in the developing mouse testis^28^. A recent study on DNA methylation of retrotransposons during male germ cell development further demonstrated that KDM5B is essential for proper spermatogenesis^39^. H3K4me2, a substrate of KDM5B, has been reported to exert an inhibitory effect on *de novo* DNA methylation that is crucial during male germ cell development. Proper regulation of DNA methylation is critical because methylation marks are removed and re-established during male germ cell development^40^, and impaired *de novo* DNA methylation of retrotransposons in gonocytes leads to apoptosis^41^. Nagamori and colleagues discovered that KDM5B colocalizes with KDM1A, which is recruited by mouse P-element-induced wimpy testis-interacting-like-4, a factor essential for recruiting *de novo* DNA methylation machinery in E16 gonocytes^39^. Furthermore, KDM5B facilitates KDM1A in removing H3K4me2 methylation marks, determining the site specificity of DNA methylation for proper differentiation of gonocytes.

#### Bone and muscle development

KDM5A and KDM5B also play key roles in the regulation of bone and muscle development. KDM5A cooperates with pRb^25^, a retinoblastoma tumor-suppressor protein and a regulator of muscle differentiation and development^42,43^. pRb is essential for cell cycle exit in myoblasts, resulting in muscle differentiation. *Rb1*^-/-^ mouse embryonic fibroblasts (MEFs) do not respond to forced expression of myogenic differentiation antigen MyoD, underscoring the functional importance of pRB in myogenesis^44^. Surprisingly, *Kdm5a* knockdown or knockout in cells defective in pRB rescues myoblast differentiation^32^. In pRB-defective cells, protein markers associated with myogenesis are re-expressed upon *Kdm5a* loss, but cell cycle withdrawal genes such as *E2f1*-3 and *cyclins* are not. H3K4me3 ChIP-seq assays using Kdm5a wild-type ESCs have shown that Kdm5a is enriched in multiple mitochondrial genes to inhibit their expression. Therefore, the inhibition of differentiation observed in *Rb*-deficient cells may be due to repression of these mitochondrial proteins by Kdm5a. These results show that removal of Kdm5a is required for activation of differentiation, allowing pRB to directly bind to and activate Kdm5a target genes with mitochondrial functions.

In addition to KDM5A, KDM5B is a key component of the epigenetic mechanism that controls osteoblast and myoblast differentiation^33^. KDM5B represses expression of Runx2/p57, a master regulator of osteoblast differentiation, by removing methylation marks on its promoter in undifferentiated, nonosteoblastic cells. As osteoblast lineage commitment increases, KDM5B is released from the *Runx2* P1 promoter and activates osteogenic differentiation. During myoblast differentiation, depletion of KDM5B leads to increased H3K4me3 and H3K27ac marks on *Runx2* promoters, facilitating Runx2/p57 transcription. Both KDM5A and KDM5B control histone methylation marks on the promoters of osteoblast or myoblast lineage-specific genes, but they do so by regulating distinct and nonoverlapping genes.

#### Angiogenesis

The development of blood vessels is always crucial for the proper functioning of cells, tissues, and organs: overgrowth of blood vessels may stimulate cancer growth, whereas blood vessel depletion may cause necrosis. Interaction between vascular endothelial growth factor A (VEGFA) and its receptor leads to vasculogenesis and angiogenesis to promote endothelial cell proliferation, migration, and permeability. Bivalent domain promoter regions carrying both activating H3K4me3 and repressive H3K27me3 marks facilitate silencing of VEGF-response genes by recruiting KDM5A^30^. KDM5A interacts with polycomb complex 2 (PRC2), which creates and maintains the repressive H3K27me3 mark. During neovascularization, recruited KDM5A accelerates inactivation of rapidly upregulated VEGF-response genes, such as *EGR3*, by removing the activating H3K4me3 mark. Moreover, KDM5A depletion enhances *EGR3* expression, the migratory ability of human umbilical vein endothelial cells (HUVECs), and cell proliferation. KDM5A serves as a brake to prevent overgrowth of blood vessels. Unlike KDM5A, which hinders angiogenesis, KDM5B promotes it. KDM5B is also highly expressed in HUVECs and is essential for endothelial angiogenic capacity in vitro and in vivo^31^. During angiogenesis, KDM5B represses *HOXA5*, an antiangiogenetic factor, in endothelial cells. HOXA5 drives endothelial cells to a resting state and inhibits angiogenesis. Overexpression of KDM5B reduces transcription of *HOXA5* by removing H3K4me3 from the TSS of *HOXA5*. Consistently, loss of KDM5B activity attenuates blood vessel growth, vascular repair, tube formation, and spheroid outgrowth cell migration. These studies support the idea that KDM5A and KDM5B exert differential effects under similar contexts via regulation of identical histone modifications.

### Dysregulation of KDM5A and KDM5B leads to developmental diseases

#### Neural diseases

As KDM5A and KDM5B play vital roles in development, their malfunction or misregulation may lead to developmental diseases^45^ (Fig. 3). Dysregulation of histone methylation contributes to the development of different neurodevelopmental diseases, such as intellectual disability (ID) syndromes and autism spectrum disorders (ASD). Among the several types of histone methylation, H3K4me3 modification via KDM5A and KDM5B is crucial for the development and function of the central nervous system^36,43,46^. Thus, mutation in or loss of the *KDM5A* and *KDM5B* genes are associated with neurodevelopmental diseases such as ASD and ID^45,47–49^. For example, a case report of a patient with autism showed that a 1.5 Mb terminal deletion of 12p13.33 encompassing 13 genes, including *KDM5A*, is associated with ASD^50^. Another case report by Han and colleagues found that KDM5A is located in the deleted region of the genes responsible for epilepsy, ID, and schizophrenia in a Korean family^51^. Other studies have also demonstrated the importance of the pathological contribution of KDM5A to neurodevelopmental diseases^48^.

KDM5B has also been observed in other studies to be an ASD candidate gene with a de novo loss-of-function (LOF) mutation^45,52^. Next-generation sequencing and exome sequencing revealed a *de novo* splicing mutation (c.283 A > G) of *KDM5B* in a patient with nonsyndromic ID^53^, six variants of KDM5B in ASD patients^52,54^, a mosaic microduplication on the chromosome region that includes *KDM5B*^55^, and a few homozygous or compound heterozygous *KDM5B* LOF mutations in patients with recessive ID^45,56^. Nevertheless, a recent study showed that *KDM5B* haploinsufficiency cannot fully explain ID and ASD in individuals with a *KDM5B* LOF variant^49^, and further molecular study of LOF *KDM5B* mutation in ID is needed. The various mutations found in *KDM5B* include missense, nonsense, and frameshift variants in key functional domains. Therefore, these mutations may impair proper recognition of specific histone modifications, disturb interaction between other epigenetic proteins or receptors, or even disable the demethylase activities of the enzymes. Although numerous studies have been performed to unravel the roles of KDM5A and KDM5B in neurodevelopmental diseases, further functional studies are required to elucidate the molecular consequences of these two lysine demethylases.

#### Heart diseases

As previously stated, KDM5A and KDM5B play crucial roles in blood vessel development^30,31^ and muscle development^25,33^. Therefore, deleterious mutations in the KDM5A and KDM5B genes may lead to cardiovascular dysfunction and other congenital heart diseases^57–60^. Congenital heart disease (CHD) is a structural abnormality of the heart that arises during embryonic development. It is the most common form of inborn malformation, affecting less than 1% of the population^61^. However, the exact pathogenesis of CHD is still poorly understood. Whole-exome sequencing performed on 30 families affected by CHD has indicated the existence of a disease-causal missense variant of KDM5A in 33% of the families^57^. In addition to these studies, other studies suggest that KDM5A and KDM5B are potential therapeutic targets for treating heart diseases^58,60^.

Approximately 20-30% of CHD survivors are at risk of experiencing neurodevelopmental disability (NDD)^59,62^. Indeed, previous research indicates that protein-truncating and deleterious missense *de novo* variants (DNVs) of certain chromatin modifiers, including *KDM5A* and *KDM5B*, account for ~40% of cases involving both CHD and ASD^63^. When Ji and colleagues analyzed 3684 CHD subjects and 1789 controls for connectome gene mutations^59^, KDM5B was included among the top 12 NDD genes with damaging DNVs in patients with CHD.

The consequences of KDM5A and KDM5B dysfunction are also observed in other developmental diseases. Accounting for the fact that KDM5 family proteins are important for the proper functioning of the testes, overexpression of KDM5A may lead to cryptorchidism^27^. The importance of proper functioning of KDM5A and KDM5B in other developmental diseases, such as osteoporosis^64,65^, obesity^13,34^, and alopecia areata^66^, has also been highlighted.

## The epigenetic role of KDM5A and KDM5B in tumorigenesis

**Fig. 4:**
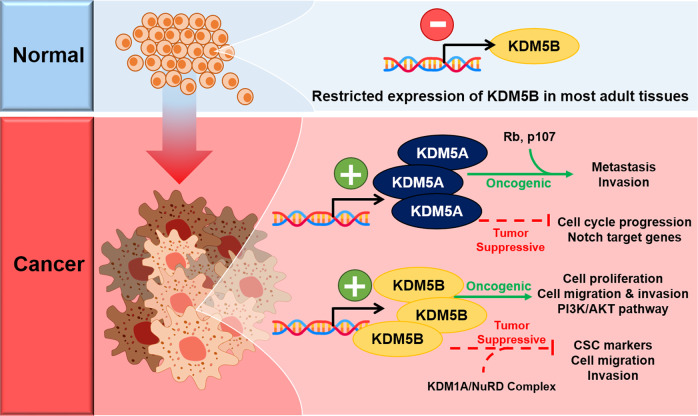
Context-dependent oncogenic and tumor-suppressive roles of KDM5A and KDM5B in breast and prostate tumorigenesis. In normal adult tissues, KDM5B expression is restricted, except in the mammary glands of pregnant females and the male reproductive organs. In contrast, most tumor tissues show increased KDM5B and KDM5A protein levels. KDM5A and KDM5B perform context-dependent oncogenic or tumor-suppressive roles in the tumorigenesis of various cancers, including breast cancer and prostate cancer. For example, KDM5A interacts with Rb or p107 to induce metastasis and invasion in breast cancer; some studies report its tumor-suppressive role by cooperating with AKT to downregulate cell cycle gene expression. KDM5B induces cell proliferation, migration, and invasion in prostate cancer by regulating the PI3K/AKT pathway but also interacts with the NuRD complex to inhibit breast cancer cell migration and invasion.

As expected from the various roles of KDM5A and KDM5B in developmental regulation, functional alterations of these two epigenetic enzymes result in tumorigenesis throughout the body (Table 1). Regulation of angiogenesis^30,31,67,68^, proliferation^24,69,70^, motility^68^, and DNA repair^71^ by these two enzymes renders them crucial for cancer progression. This review mainly characterizes the roles of KDM5A and KDM5B in breast cancer (BC) and prostate cancer (PC) because previous studies have shown that normal expression of KDM5B is highly restricted in adult tissues except in the mammary glands of pregnant females and the male reproductive organ, the testis^70,72–74^. Although no studies indicate differential expression of KDM5A in the mammary glands or testis, KDM5A is well known for its role in various cancers. Therefore, the oncogenic roles of KDM5A and KDM5B in BC and PC is covered below (Fig. 4).

**Table 1:** Differential levels of KDM5A and KDM5B in various cancers.

Cancer Type	KDM5A	KDM5B
Acute myeloid leukemia	Upregulated^101^	Upregulated^68^
Breast cancer	Upregulated^76,80,100^	Upregulated^69,102,103^
Bladder cancer	N/A^a^	Upregulated^104^
Colon cancer	N/A	Upregulated^105^
Ewing sarcoma	Upregulated^106^	N/A
Gastric cancer	Upregulated^107^	Upregulated^108^
Glioblastoma	Upregulated^109,110^ Downregulated^111^	Upregulated^112,113^
Head and neck cancer	Upregulated^114,115^	Upregulated^116,117^
Hepatocellular carcinoma	Upregulated^67^	Upregulated^118^
Lung cancer	Upregulated^119,120^	Upregulated^121^
Melanoma	Downregulated^122^	Upregulated^123,124^
Multiple myeloma	Upregulated^125^	Upregulated^95^
Osteosarcoma	Upregulated^11^	Upregulated^126^
Ovarian cancer	Upregulated^127^	Upregulated^128^
Pancreas cancer	Upregulated^129^	Upregulated^130^
Prostate cancer	Upregulated^88,93^	Upregulated^90,91^
Renal carcinoma	Upregulated^131^	Upregulated^131^

^a^*N/A* not available.

### The roles of KDM5A and KDM5B in breast cancer

Of the various epigenetic histone modifiers, KDM5A and KDM5B are overexpressed in different forms of BC^69,72–78^. KDM5A is amplified in approximately 15% of BC^76^. In vivo metastasis models also show that KDM5A knockdown or inhibition reduces BC metastasis to the lungs^77^. Surprisingly, contrary to the prevailing notion that KDM5A-mediated H3K4me3 removal controls target gene repression, a catalytically dead KDM5A variant also promotes BC metastasis and invasion. In this case, KDM5A recruits and binds to Rb or p107 to activate its target genes to promote metastasis. Parallel to the role of KDM5A in BC progression, KDM5B has also been confirmed to promote BC growth by inducing cell cycle progression by indirectly activating expression of cyclin D1, a cell cycle stimulator^79^. KDM5B removes H3K4me3 and suppresses *let-7e* microRNA, which acts as a tumor suppressor to downregulate cyclin D1. These mechanisms highlight the oncogenic potential of dysregulated KDM5A and KDM5B in the context of cell cycle gene regulation.

However, some studies also point out the tumor-suppressive roles of KDM5A in BC. One study reported that KDM5A cooperates with AKT to downregulate cell cycle gene expression in advanced-stage BC^80^. KDM5A may function as a tumor suppressor by removing H3K4me3 on cell cycle-promoting genes. Such contradictory results regarding the roles of BC progression highlight the pleiotropic roles of epigenetic regulators and also suggest the need for deeper study to fully understand their molecular mechanism under certain conditions.

In line with its importance in regulating cell differentiation, improper functioning of KDM5B is associated with different tumorigenic abilities in subtype-specific types of BC. For instance, KDM5B is highly associated with estrogen-receptor-positive (ER^+^) cell lines^70,73,74^. KDM5B knockdown pronouncedly inhibits estradiol (E2)-dependent tumor growth of ER^+^ breast cancer cells^70^. Similarly, another study showed that KDM5B drives carcinogenesis in luminal lineage ER^+^ breast tumors by associating with the CTCF transcription factor^78^. In addition to its role in ER^+^ BCs, KDM5B contributes to BC progression in triple-negative breast cancer (TNBC), a highly aggressive subtype of breast cancer defined by a lack of ER, progesterone receptor, and human epidermal growth factor receptor 2 (HER2) expression^81^. Silencing KDM5B markedly reduces the migration and invasive potential of TNBC cells by regulating expression of lncRNA *MALAT1* and its metastasis-associated target genes^82^. Although numerous studies have shown the importance of KDM5B in subtype-specific BC tumorigenesis, more research needs to be conducted on KDM5A to uncover the correlation between KDM5A and subtype-specific BC growth.

Despite several studies reporting evidence for the oncogenic roles of KDM5A and KDM5B in the development of BC, some studies provide contradictory results that indicate that high expression of KDM5A is associated with a better prognosis in BC^83^. It is not uncommon that a transcriptional regulator has context-dependent oncogenic and tumor-suppressing functions. According to one study, KDM5A inactivates Myc activity^84^ and directly interacts with recombination signal binding protein-J to function as a tumor suppressor by removing the H3K4me3 activation mark at Notch target genes^85^. Tumor-suppressive roles of KDM5B in BC have also been suggested^86^. In TNBC, suppression of KDM5B increases expression of the cancer stem cell markers *Sox2* and *Nanog*. This aligns with the fact that KDM5B binds to the promoters of and regulates expression of core pluripotency regulators in mESCs^46^. Other studies also demonstrate that overexpression of KDM5B inhibits BC cell migration and invasion by cooperating with the KDM1A/nucleosome remodeling and deacetylase (NuRD) complex^68,87^. Such conflicting reports on the tumorigenic roles of KDM5A and KDM5B may be attributed to the various target genes and interacting partners that play diverse roles in regulation of the cell cycle, development, and differentiation (Table 2). Further studies are needed to clarify the roles of these two epigenetic proteins not only in BC carcinogenesis but also in other diseases.

**Table 2:** Interacting partners of KDM5A and KDM5B.

Protein	Interacting partner	Function	Reference
KDM5A	NR^a^ (e.g., ER^b^, AR^c^)	NR-mediated transcription	^132^
	p50	NK-cell activity	^133^
	PRC2^d^ (Suz12)	Regulation of developmental genes	^134^
	NuRD^e^ complex		^135^
	SIN3B^f^-HDAC^g^		
	Retinoblastoma (RB)	Cellular senescence	^136^
	TBP^h^	Early development	
	p107	Tumorigenesis	
	Rhombotin-2	T-cell leukemogenesis	^137^
KDM5B	PIWIL4^i^	DNA methylation of retrotransposons	^138^
	CBX4^j^	Transcriptional repression	^39^
	HDAC1 (NuRD complex)		^12^
	Retinoblastoma (RB)	Cellular senescence	^139^
	PAX9^k^	Early embryonic development in mouse (craniofacial features, limbs, teeth, and thymus)	^140^
	FOXG1b^l^ (BF-1)	Early embryonic development in mouse (brain)	
	NR (e.g., ER, AR)	Tumorigenesis	^70,89^
	HDAC4	Differentiation of mouse mammary glands and breast cancer development	^73^

^a^*NR* nuclear receptor.

^b^*ER* estrogen receptor.

^c^*AR* androgen receptor.

^d^*PRC2* polycomb repressive complex 2.

^e^*NuRD* nucleosome remodeling deacetylase.

^f^*SIN3B* SIN3 transcription regulator family member B.

^g^*HDAC* histone deacetylase.

^h^*TBP* TATA-box-binding protein.

^i^*PIWIL-4* Piwi-like protein 4.

^j^*CBX4* chromobox 4.

^k^*PAX9* paired Box 9.

^l^*FOXG1b* forkhead Box G1b.

### The roles of KDM5A and KDM5B in prostate cancer

Many studies have reported the importance of KDM5A and KDM5B for PC growth. KDM5A and KDM5B perform oncogenic functions in PC and are significantly upregulated in PC samples compared to normal prostate samples^88,89^. As previously mentioned, both KDM5A and KDM5B are important in spermatogenesis^27,28^. Therefore, the roles of KDM5A and KDM5B upon their deregulation in the context of PC tumorigenesis are discussed below (Fig. 4).

KDM5B is upregulated in PC tissues compared to benign prostate samples^89^. KDM5B regulates and interacts with androgen receptor, which is crucial for PC progression. Additional studies also demonstrate that depletion of KDM5B reduces PC proliferation, cell cycle progression, and migration^90,91^. Deeper molecular studies using KDM5B knockout mouse models and biochemistry methods have shown that KDM5B governs and is governed by the PI3K/AKT pathway, which is crucial for PC progression^91,92^. Specifically, loss of KDM5B abrogates P110α and PIP3 levels and therefore weakens the PI3K/AKT signaling pathway, attenuating the tumorigenesis of PC^91^. On the other hand, the PI3K/AKT pathway has been shown to act upstream of KDM5B. AKT inhibition reduces global H3K4 methylation levels by regulating *miR-137* to transcriptionally repress KDM5B expression, which suppresses PC growth^92^. All this evidence supports the oncogenic role of KDM5B in PC progression, suggesting KDM5B as a potential target for PC.

Although a plethora of studies have contributed to our understanding of the role of KDM5B in PC progression, only a few studies report on the role of KDM5A in PC progression. Altered expression levels of KDM5A in PC have been observed^88,93^. Compared to that of normal prostate tissues, the mRNA level of *KDM5A* is elevated in prostate tumor tissues^88^. Another study demonstrated that overexpression of KDM5A induces PC cell growth, migration, and invasion^93^. In vivo studies using xenograft mouse models also show increased tumor sizes upon KDM5A overexpression. KDM5A has been reported to be elevated in PC, but the exact molecular mechanism of KDM5A in PC progression remains to be elucidated. Accumulating evidence supports that KDM5A and KDM5B family proteins act as protumorigenic factors in PC. Nevertheless, more in-depth research is necessary to solidify the relationship between PC and the KDM5A and KDM5B proteins.

### Current inhibitors of KDM5

Small molecules that specifically inhibit KDM5 family proteins have been identified through high-throughput screening. Of the various KDM5 inhibitors, PBIT, KDM5-C49, KDM5-C70, GDK467, KDOAM-25, CPI-455, KDM5-Inh1, and KDM5-Inh1A^94–97^, none specifically inhibit KDM5A or KDM5B individually. This lack of specific inhibitors of KDM5A or KDM5B may be attributed to the common domains and structures of KDM5 proteins. KDM5 proteins depend on JmjC domains for their catalytic activity, and this domain is highly conserved among the KDM5 orthologs of different species. The question is whether individually specific KDM5 demethylase inhibitors can be developed that do not target the other proteins. GS-5801 by Gilead Sciences was under evaluation for its safety and tolerability in a phase 1b clinical trial for hepatitis B virus-infected patients, but the study was terminated in 2018^98^. Further studies are necessary to break through the current barriers in the development of KDM5A- or KDM5B-selective inhibitors.

An in vitro and in vivo preclinical study showed that pharmacological inhibition of KDM5 via KDM5-Inh1 developed by Gilead Sciences results in antitumor effects in HER2^+^ BC cell lines^97^. KDM5-Inh1, a KDM5 inhibitor, shows the highest inhibitory activity on KDM5B at an IC_50_ value of 0.28 nM. KDM5-Inh1 also targets KDM5A at a higher concentration, with an IC_50_ value of 4.3 nM. KDM5-Inh1 demonstrates antitumor effects in HER2^+^ BC cells by inducing cell cycle inhibition and apoptosis. Additional in vivo experiments using KDM5-Inh1A, a close structural analog to KDM5-Inh1, revealed reduced tumor formation and tumor growth. Furthermore, KDM5-Inh1 treatment in HER2^+^ BC cells resistant to the HER2-targeting agent trastuzumab reduced proliferation in both sensitive HER2^+^ BC cells and trastuzumab-resistant HER2^+^ BC cells.

Organometallic compounds based on transition metals such as rhodium (Rh) and iridium are emerging as promising scaffolds for antitumor agents. Rh(III) complex 1 inhibits KDM5A activity at an IC_50_ value of 23.2 nM and suppresses BC cell growth in vivo without showing significant toxicity^99,100^. Nonetheless, further studies are required to confirm the specificity of Rh(III) complex 1 on KDM5A over KDM5B, KDM5C, KDM5D, or other lysine demethylases in other cancers to fully assess their potential in cancer treatment. More studies are required to develop inhibitors that individually target proteins within the KDM5 protein family. A vast number of preclinical studies have shown the potential of KDM5A and KDM5B as therapeutic targets, and further investigations aiming to develop specific inhibitors for these proteins will facilitate the treatment of various diseases, including cancer.

## Discussion

Mounting evidence supports that KDM5 demethylases are pivotal in development and cancer progression. KDM5A and KDM5B play important roles in various physiological and pathological events ranging from the maintenance of homeostasis to the development of cancer. Most epigenetic modulators in humans can recognize several epigenetic marks, and many of these epigenetic marks are also recognized by other members within the protein family^2^. This innate characteristic of epigenetic enzymes makes it difficult to identify the exact molecular mechanisms that lead to certain physiological effects. In the same sense, KDM5A and KDM5B both remove di- or trimethylation marks of the same histone modification, but clear reasons why each protein has different effects have yet to be discovered. Of the many possibilities, the final physiological functions carried out by these enzymes may depend on cellular contexts and interacting partners. The contradictory functions of proteins in the same family with the same enzymatic activity suggest the need for extensive research to identify the exact molecular mechanism responsible for their distinct physiological functions. There is a need for further studies to elucidate the reasons for these differences among epigenetic proteins that is not limited to KDM5s.

Efforts to develop KDM5A-specific or KDM5B-specific inhibitors also emphasize the idea that KDM5A and KDM5B do not always function to give rise to the same biological effects, providing a new viewpoint to see them as individual proteins. Regardless, the development of individually specific KDM5 inhibitors will not be easy, and several challenges must be overcome. As mentioned above, KDM5 proteins share highly similar catalytic domains with each other and other lysine demethylases. Therefore, inhibitors targeting the catalytic domains of KDM5 enzymes lead to unwanted off-target effects on other enzymes. Perhaps targeting the unique allosteric sites in each protein is an alternative solution to overcome such a problem. Additionally, the heterogenic roles of KDM5A and KDM5B in different cancers and developmental stages make optimization of these inhibitors difficult. Although overexpression of KDM5A and KDM5B in several cancers has been confirmed and their oncogenic pathways have been identified, some studies report on the context-dependent tumor-suppressive roles of KDM5A and KDM5B. In this case, hampering the protein‒protein interaction between KDM5A or KDM5B and its partner protein under different contexts may improve the efficacy of the inhibitors developed. Although the functional ambiguity of KDM5A and KDM5B may slow the development of effective inhibitors, their diverse functions underscore their potential as cancer biomarkers and drug targets. Additionally, unveiling the molecular mechanism behind the ambiguous roles of KDM5A and KDM5B will act as a basis for the development of personalized treatment methods for patients with cancer and other diseases. Overall, a better understanding of the intertwined biochemistry underlying various physiological functions regulated by KDM5A and KDM5B will fill in the gaps of their story as potential epigenetic modulators and therapeutic targets.

## References

[1] Peterson CL, Laniel MA (2004). Histones and histone modifications. Curr. Biol..

[2] Bannister AJ, Kouzarides T (2011). Regulation of chromatin by histone modifications. Cell Res..

[3] Rea S (2000). Regulation of chromatin structure by site-specific histone H3 methyltransferases. Nature.

[4] Paik WK, Kim S (1973). Enzymatic demethylation of calf thymus histones. Biochem. Biophys. Res. Commun..

[5] Peters AH (2001). Loss of the Suv39h histone methyltransferases impairs mammalian heterochromatin and genome stability. Cell.

[6] Pasini D, Bracken AP, Jensen MR, Lazzerini Denchi E, Helin K (2004). Suz12 is essential for mouse development and for EZH2 histone methyltransferase activity. EMBO J..

[7] Blair LP, Cao J, Zou MR, Sayegh J, Yan Q (2011). Epigenetic regulation by lysine demethylase 5 (KDM5) enzymes in cancer. Cancers (Basel).

[8] Jangravi Z (2015). Two splice variants of Y chromosome-located lysine-specific demethylase 5D have distinct function in prostate cancer cell line (DU-145). J. Proteome Res..

[9] Jensen LR (2005). Mutations in the JARID1C gene, which is involved in transcriptional regulation and chromatin remodeling, cause X-linked mental retardation. Am. J. Hum. Genet..

[10] Yang GJ (2021). The emerging role of KDM5A in human cancer. J. Hematol. Oncol..

[11] Peng D (2021). Histone demethylase KDM5A promotes tumorigenesis of osteosarcoma tumor. Cell Death Discov..

[12] Klein BJ (2014). The histone-H3K4-specific demethylase KDM5B binds to its substrate and product through distinct PHD fingers. Cell Rep..

[13] Brier AB (2017). The KDM5 family is required for activation of pro-proliferative cell cycle genes during adipocyte differentiation. Nucleic Acids Res.

[14] Liu X, Secombe J (2015). The histone demethylase KDM5 activates gene expression by recognizing chromatin context through Its PHD reader motif. Cell Rep..

[15] Lange C, Calegari F (2010). Cdks and cyclins link G1 length and differentiation of embryonic, neural and hematopoietic stem cells. Cell Cycle.

[16] Calder A (2013). Lengthened G1 phase indicates differentiation status in human embryonic stem cells. Stem Cells Dev..

[17] Coronado D (2013). A short G1 phase is an intrinsic determinant of naive embryonic stem cell pluripotency. Stem Cell Res..

[18] Beshiri ML (2012). Coordinated repression of cell cycle genes by KDM5A and E2F4 during differentiation. Proc. Natl Acad. Sci. USA.

[19] Lopez-Bigas N (2008). Genome-wide analysis of the H3K4 histone demethylase RBP2 reveals a transcriptional program controlling differentiation. Mol. Cell.

[20] Christensen J (2007). RBP2 belongs to a family of demethylases, specific for tri-and dimethylated lysine 4 on histone 3. Cell.

[21] Pasini D (2008). Coordinated regulation of transcriptional repression by the RBP2 H3K4 demethylase and polycomb-repressive complex 2. Genes Dev..

[22] Ohguchi, Y. & Ohguchi, H. Diverse functions of KDM5 in cancer: transcriptional repressor or activator? *Cancers (Basel)* **14**, 3270 (2022). 10.3390/cancers14133270 PMC9265395 35805040

[23] Dey BK (2008). The histone demethylase KDM5b/JARID1b plays a role in cell fate decisions by blocking terminal differentiation. Mol. Cell Biol..

[24] Yamane K (2007). PLU-1 is an H3K4 demethylase involved in transcriptional repression and breast cancer cell proliferation. Mol. Cell.

[25] Benevolenskaya EV, Murray HL, Branton P, Young RA, Kaelin WG (2005). Binding of pRB to the PHD protein RBP2 promotes cellular differentiation. Mol. Cell.

[26] Kong SY, Kim W, Lee HR, Kim HJ (2018). The histone demethylase KDM5A is required for the repression of astrocytogenesis and regulated by the translational machinery in neural progenitor cells. FASEB J..

[27] Nishio H (2014). Distinctive changes in histone H3K4 modification mediated via Kdm5a expression in spermatogonial stem cells of cryptorchid testes. J. Urol..

[28] Madsen B (2003). PLU-1, a transcriptional repressor and putative testis-cancer antigen, has a specific expression and localisation pattern during meiosis. Chromosoma.

[29] Cui Y (2018). Pig KDM5B: mRNA expression profiles of different tissues and testicular cells and association analyses with testicular morphology traits. Gene.

[30] Chen J (2020). Two faces of bivalent domain regulate VEGFA responsiveness and angiogenesis. Cell Death Dis..

[31] Fork C (2015). Epigenetic Regulation of Angiogenesis by JARID1B-Induced Repression of HOXA5. Arterioscler. Thromb. Vasc. Biol..

[32] Varaljai R (2015). Increased mitochondrial function downstream from KDM5A histone demethylase rescues differentiation in pRB-deficient cells. Genes Dev..

[33] Rojas A (2015). Epigenetic control of the bone-master Runx2 gene during osteoblast-lineage commitment by the histone demethylase JARID1B/KDM5B. J. Biol. Chem..

[34] Guo L, Guo YY, Li BY, Peng WQ, Tang QQ (2019). Histone demethylase KDM5A is transactivated by the transcription factor C/EBPbeta and promotes preadipocyte differentiation by inhibiting Wnt/beta-catenin signaling. J. Biol. Chem..

[35] Gage FH (2000). Mammalian neural stem cells. Science.

[36] Schmitz SU (2011). Jarid1b targets genes regulating development and is involved in neural differentiation. EMBO J..

[37] Kubota H, Brinster RL (2018). Spermatogonial stem cells. Biol. Reprod..

[38] Simpson AJ, Caballero OL, Jungbluth A, Chen YT, Old LJ (2005). Cancer/testis antigens, gametogenesis and cancer. Nat. Rev. Cancer.

[39] Nagamori I (2018). Relationship between PIWIL4-Mediated H3K4me2 Demethylation and piRNA-dependent DNA methylation. Cell Rep..

[40] Kobayashi H (2013). High-resolution DNA methylome analysis of primordial germ cells identifies gender-specific reprogramming in mice. Genome Res..

[41] Aravin AA, Sachidanandam R, Girard A, Fejes-Toth K, Hannon GJ (2007). Developmentally regulated piRNA clusters implicate MILI in transposon control. Science.

[42] Zacksenhaus E (1996). pRb controls proliferation, differentiation, and death of skeletal muscle cells and other lineages during embryogenesis. Genes Dev..

[43] de Bruin A (2003). Rb function in extraembryonic lineages suppresses apoptosis in the CNS of Rb-deficient mice. Proc. Natl Acad. Sci. USA.

[44] Novitch BG, Mulligan GJ, Jacks T, Lassar AB (1996). Skeletal muscle cells lacking the retinoblastoma protein display defects in muscle gene expression and accumulate in S and G2 phases of the cell cycle. J. Cell Biol..

[45] Faundes V (2018). Histone lysine methylases and demethylases in the landscape of human developmental disorders. Am. J. Hum. Genet..

[46] Kidder BL, Hu G, Zhao K (2014). KDM5B focuses H3K4 methylation near promoters and enhancers during embryonic stem cell self-renewal and differentiation. Genome Biol..

[47] Najmabadi H (2011). Deep sequencing reveals 50 novel genes for recessive cognitive disorders. Nature.

[48] Vallianatos CN, Iwase S (2015). Disrupted intricacy of histone H3K4 methylation in neurodevelopmental disorders. Epigenomics.

[49] Lebrun N (2018). Novel KDM5B splice variants identified in patients with developmental disorders: Functional consequences. Gene.

[50] Silva IM, Rosenfeld J, Antoniuk SA, Raskin S, Sotomaior VS (2014). A 1.5Mb terminal deletion of 12p associated with autism spectrum disorder. Gene.

[51] Han, J. Y. & Park, J. Variable phenotypes of epilepsy, intellectual disability, and schizophrenia caused by 12p13.33-p13.32 terminal microdeletion in a Korean family: a case report and literature review. *Genes (Basel)* **12**, 1001 (2021). 10.3390/genes12071001 PMC8303811 34210021

[52] Iossifov I (2014). The contribution of de novo coding mutations to autism spectrum disorder. Nature.

[53] Athanasakis E (2014). Next generation sequencing in nonsyndromic intellectual disability: from a negative molecular karyotype to a possible causative mutation detection. Am. J. Med. Genet. A.

[54] De Rubeis S (2014). Synaptic, transcriptional and chromatin genes disrupted in autism. Nature.

[55] Miolo G (2019). A novel mosaic 1q32.1 microduplication identified through chromosome microarray analysis: narrowing the smallest critical region including KDM5B gene found associated with neurodevelopmetal disorders. Eur. J. Med. Genet..

[56] Martin HC (2018). Quantifying the contribution of recessive coding variation to developmental disorders. Science.

[57] Szot JO (2018). A Screening approach to identify clinically actionable variants causing congenital heart disease in exome data. Circ. Genom. Precis. Med..

[58] Zhou W (2020). Notch1-mediated histone demethylation of HCN4 contributes to aconitine-induced ventricular myocardial dysrhythmia. Toxicol. Lett..

[59] Ji W (2020). De novo damaging variants associated with congenital heart diseases contribute to the connectome. Sci. Rep..

[60] Li Y (2018). Histone demethylase JARID1B regulates proliferation and migration of pulmonary arterial smooth muscle cells in mice with chronic hypoxia-induced pulmonary hypertension via nuclear factor-kappa B (NFkB). Cardiovasc. Pathol..

[61] van der Linde D (2011). Birth prevalence of congenital heart disease worldwide: a systematic review and meta-analysis. J. Am. Coll. Cardiol..

[62] Laraja K (2017). Neurodevelopmental outcome in children after fetal cardiac intervention for aortic stenosis with evolving hypoplastic left heart syndrome. J. Pediatr..

[63] Jin SC (2017). Contribution of rare inherited and de novo variants in 2,871 congenital heart disease probands. Nat. Genet..

[64] Wang C (2016). KDM5A controls bone morphogenic protein 2-induced osteogenic differentiation of bone mesenchymal stem cells during osteoporosis. Cell Death Dis..

[65] Zhang X (2020). Extracellular vesicle-encapsulated miR-29b-3p released from bone marrow-derived mesenchymal stem cells underpins osteogenic differentiation. Front. Cell Dev. Biol..

[66] Zhao M (2012). Abnormal epigenetic modifications in peripheral blood mononuclear cells from patients with alopecia areata. Br. J. Dermatol.

[67] Ma YS (2021). KDM5A silencing transcriptionally suppresses the FXYD3-PI3K/AKT axis to inhibit angiogenesis in hepatocellular cancer via miR-433 up-regulation. J. Cell Mol. Med..

[68] Li Q (2011). Binding of the JmjC demethylase JARID1B to LSD1/NuRD suppresses angiogenesis and metastasis in breast cancer cells by repressing chemokine CCL14. Cancer Res..

[69] Zhang ZG (2019). KDM5B promotes breast cancer cell proliferation and migration via AMPK-mediated lipid metabolism reprogramming. Exp. Cell Res..

[70] Catchpole S (2011). PLU-1/JARID1B/KDM5B is required for embryonic survival and contributes to cell proliferation in the mammary gland and in ER+ breast cancer cells. Int. J. Oncol..

[71] Mocavini I (2019). JARID1B expression and its function in DNA damage repair are tightly regulated by miRNAs in breast cancer. Cancer Sci..

[72] Madsen B (2002). Characterisation and developmental expression of mouse Plu-1, a homologue of a human nuclear protein (PLU-1) which is specifically up-regulated in breast cancer. Gene Expr. Patterns.

[73] Barrett A (2007). Breast cancer associated transcriptional repressor PLU-1/JARID1B interacts directly with histone deacetylases. Int. J. Cancer.

[74] Lu PJ (1999). A novel gene (PLU-1) containing highly conserved putative DNA/chromatin binding motifs is specifically up-regulated in breast cancer. J. Biol. Chem..

[75] Barrett A (2002). PLU-1 nuclear protein, which is upregulated in breast cancer, shows restricted expression in normal human adult tissues: a new cancer/testis antigen?. Int. J. Cancer.

[76] Hou J (2012). Genomic amplification and a role in drug-resistance for the KDM5A histone demethylase in breast cancer. Am. J. Transl. Res..

[77] Cao J (2014). Histone demethylase RBP2 is critical for breast cancer progression and metastasis. Cell Rep..

[78] Yamamoto S (2014). JARID1B is a luminal lineage-driving oncogene in breast cancer. Cancer Cell.

[79] Mitra D, Das PM, Huynh FC, Jones FE (2011). Jumonji/ARID1 B (JARID1B) protein promotes breast tumor cell cycle progression through epigenetic repression of microRNA let-7e. J. Biol. Chem..

[80] Spangle JM (2016). PI3K/AKT signaling regulates H3K4 methylation in breast cancer. Cell Rep..

[81] Foulkes WD, Smith IE, Reis-Filho JS (2010). Triple-negative breast cancer. N. Engl. J. Med..

[82] Bamodu OA (2016). Aberrant KDM5B expression promotes aggressive breast cancer through MALAT1 overexpression and downregulation of hsa-miR-448. BMC Cancer.

[83] Paolicchi E, Crea F, Farrar WL, Green JE, Danesi R (2013). Histone lysine demethylases in breast cancer. Crit. Rev. Oncol. Hematol..

[84] Secombe J, Eisenman RN (2007). The function and regulation of the JARID1 family of histone H3 lysine 4 demethylases: the Myc connection. Cell Cycle.

[85] Liefke R (2010). Histone demethylase KDM5A is an integral part of the core Notch-RBP-J repressor complex. Genes Dev..

[86] Yeh IJ (2019). Phosphorylation of the histone demethylase KDM5B and regulation of the phenotype of triple negative breast cancer. Sci. Rep..

[87] Cariou S, Catzavelos C, Slingerland JM (1998). Prognostic implications of expression of the cell cycle inhibitor protein p27Kip1. Breast Cancer Res. Treat..

[88] Vieira FQ (2014). Deregulated expression of selected histone methylases and demethylases in prostate carcinoma. Endocr. Relat. Cancer.

[89] Xiang Y (2007). JARID1B is a histone H3 lysine 4 demethylase up-regulated in prostate cancer. Proc. Natl Acad. Sci. USA.

[90] Yang Z, Xu JX, Fang DP, Ke J (2020). Analysis of key genes reveal lysine demethylase 5B promotes prostate cancer progression. Oncol. Lett..

[91] Li G (2020). KDM5B is essential for the hyperactivation of PI3K/AKT signaling in prostate tumorigenesis. Cancer Res..

[92] Khan MI (2019). AKT inhibition modulates H3K4 demethylase levels in PTEN-null prostate cancer. Mol. Cancer Ther..

[93] Du C, Lv C, Feng Y, Yu S (2020). Activation of the KDM5A/miRNA-495/YTHDF2/m6A-MOB3B axis facilitates prostate cancer progression. J. Exp. Clin. Cancer Res..

[94] Johansson C (2016). Structural analysis of human KDM5B guides histone demethylase inhibitor development. Nat. Chem. Biol..

[95] Tumber A (2017). Potent and selective KDM5 inhibitor stops cellular demethylation of H3K4me3 at transcription start sites and proliferation of MM1S myeloma cells. Cell Chem. Biol..

[96] Vinogradova M (2016). An inhibitor of KDM5 demethylases reduces survival of drug-tolerant cancer cells. Nat. Chem. Biol..

[97] Paroni G (2019). HER2-positive breast-cancer cell lines are sensitive to KDM5 inhibition: definition of a gene-expression model for the selection of sensitive cases. Oncogene.

[98] Gilmore S (2017). Antiviral activity of GS-5801, a liver-targeted prodrug of a lysine demethylase 5 inhibitor, in a hepatitis B virus primary human hepatocyte infection model. J. Hepatol..

[99] Yang GJ (2018). Selective inhibition of lysine-specific demethylase 5A (KDM5A) using a rhodium(III) complex for triple-negative breast cancer therapy. Angew. Chem. Int. Ed. Engl..

[100] Yang, G. J., Ko, C. N., Zhong, H. J., Leung, C. H. & Ma, D. L. Structure-based discovery of a selective KDM5A inhibitor that exhibits anti-cancer activity via inducing cell cycle arrest and senescence in breast cancer cell lines. *Cancers (Basel)* **11**, 92 (2019). 10.3390/cancers11010092 PMC6360022 30650517

[101] Choi, H. J. et al. Role of RBP2-induced ER and IGF1R-ErbB signaling in tamoxifen resistance in breast cancer. *J. Natl. Cancer Inst*. **110**10.1093/jnci/djx207 (2018). 10.1093/jnci/djx207 29028222

[102] Coleman JA (2011). T cells reactive with HLA-A*0201 peptides from the histone demethylase JARID1B are found in the circulation of breast cancer patients. Int. J. Cancer.

[103] Zhao LH, Liu HG (2015). Immunohistochemical detection and clinicopathological significance of JARID1B/KDM5B and P16 expression in invasive ductal carcinoma of the breast. Genet. Mol. Res..

[104] Hayami S (2010). Overexpression of the JmjC histone demethylase KDM5B in human carcinogenesis: involvement in the proliferation of cancer cells through the E2F/RB pathway. Mol. Cancer.

[105] Ohta K (2013). Depletion of JARID1B induces cellular senescence in human colorectal cancer. Int. J. Oncol..

[106] McCann TS (2020). KDM5A and PHF2 positively control expression of pro-metastatic genes repressed by EWS/Fli1, and promote growth and metastatic properties in Ewing sarcoma. Oncotarget.

[107] Zeng J (2010). The histone demethylase RBP2 Is overexpressed in gastric cancer and its inhibition triggers senescence of cancer cells. Gastroenterology.

[108] Li Y (2019). NEK2 promotes proliferation, migration and tumor growth of gastric cancer cells via regulating KDM5B/H3K4me3. Am. J. Cancer Res..

[109] Banelli B (2015). The histone demethylase KDM5A is a key factor for the resistance to temozolomide in glioblastoma. Cell Cycle.

[110] Romani, M., Daga, A., Forlani, A., Pistillo, M. P. & Banelli, B. Targeting of histone demethylases KDM5A and KDM6B inhibits the proliferation of temozolomide-resistant glioblastoma cells. *Cancers (Basel)* **11**, 878 (2019). 10.3390/cancers11060878 PMC6627323 31238504

[111] Dai B (2018). Histone demethylase KDM5A inhibits glioma cells migration and invasion by down regulating ZEB1. Biomed. Pharmacother..

[112] Dai B (2014). Overexpressed KDM5B is associated with the progression of glioma and promotes glioma cell growth via downregulating p21. Biochem. Biophys. Res. Commun..

[113] Fang L (2016). Jumonji AT-rich interactive domain 1B overexpression is associated with the development and progression of glioma. Int. J. Mol. Med..

[114] You JH, Lee J, Roh JL (2021). Mitochondrial pyruvate carrier 1 regulates ferroptosis in drug-tolerant persister head and neck cancer cells via epithelial-mesenchymal transition. Cancer Lett..

[115] Li H (2014). Genomic analysis of head and neck squamous cell carcinoma cell lines and human tumors: a rational approach to preclinical model selection. Mol. Cancer Res..

[116] Huang D (2018). KDM5B overexpression predicts a poor prognosis in patients with squamous cell carcinoma of the head and neck. J. Cancer.

[117] Cui Z (2015). PLU-1/JARID1B overexpression predicts proliferation properties in head and neck squamous cell carcinoma. Oncol. Rep..

[118] Wang D (2016). Depletion of histone demethylase KDM5B inhibits cell proliferation of hepatocellular carcinoma by regulation of cell cycle checkpoint proteins p15 and p27. J. Exp. Clin. Cancer Res..

[119] Oser MG (2019). The KDM5A/RBP2 histone demethylase represses NOTCH signaling to sustain neuroendocrine differentiation and promote small cell lung cancer tumorigenesis. Genes Dev..

[120] Teng YC (2013). Histone demethylase RBP2 promotes lung tumorigenesis and cancer metastasis. Cancer Res..

[121] Kuo KT (2018). Histone demethylase JARID1B/KDM5B promotes aggressiveness of non-small cell lung cancer and serves as a good prognostic predictor. Clin. Epigenetics.

[122] Roesch A (2005). Retinoblastoma-binding protein 2-homolog 1: a retinoblastoma-binding protein downregulated in malignant melanomas. Mod. Pathol..

[123] Kuzbicki L, Lange D, Straczynska-Niemiec A, Chwirot BW (2013). JARID1B expression in human melanoma and benign melanocytic skin lesions. Melanoma Res.

[124] Roesch A (2008). RBP2-H1/JARID1B is a transcriptional regulator with a tumor suppressive potential in melanoma cells. Int. J. Cancer.

[125] Ohguchi H (2021). Lysine Demethylase 5A is Required for MYC Driven Transcription in Multiple Myeloma. Blood Cancer Discov..

[126] Wang W, Zheng K, Pei Y, Zhang X (2018). Histone demethylase JARID1B is overexpressed in osteosarcoma and upregulates cyclin D1 expression via demethylation of H3K27me3. Oncol. Res..

[127] Feng T, Wang Y, Lang Y, Zhang Y (2017). KDM5A promotes proliferation and EMT in ovarian cancer and closely correlates with PTX resistance. Mol. Med. Rep..

[128] Wang L, Mao Y, Du G, He C, Han S (2015). Overexpression of JARID1B is associated with poor prognosis and chemotherapy resistance in epithelial ovarian cancer. Tumour Biol..

[129] Lin W (2015). Dynamic epigenetic regulation by menin during pancreatic islet tumor formation. Mol. Cancer Res..

[130] Shen X (2018). Jumonji AT-rich interactive domain 1B promotes the growth of pancreatic tumors via the phosphatase and tensin homolog/protein kinase B signaling pathway. Oncol. Lett..

[131] Kumar A (2019). Reduction in H3K4me patterns due to aberrant expression of methyltransferases and demethylases in renal cell carcinoma: prognostic and therapeutic implications. Sci. Rep..

[132] Chan SW, Hong W (2001). Retinoblastoma-binding protein 2 (Rbp2) potentiates nuclear hormone receptor-mediated transcription. J. Biol. Chem..

[133] Zhao D (2016). H3K4me3 demethylase Kdm5a is required for NK cell activation by associating with p50 to suppress SOCS1. Cell Rep..

[134] Peng JC (2009). Jarid2/Jumonji coordinates control of PRC2 enzymatic activity and target gene occupancy in pluripotent cells. Cell.

[135] Nishibuchi G (2014). Physical and functional interactions between the histone H3K4 demethylase KDM5A and the nucleosome remodeling and deacetylase (NuRD) complex. J. Biol. Chem..

[136] Kim YW, Otterson GA, Kratzke RA, Coxon AB, Kaye FJ (1994). Differential specificity for binding of retinoblastoma binding protein 2 to RB, p107, and TATA-binding protein. Mol. Cell Biol..

[137] Mao S, Neale GA, Goorha RM (1997). T-cell oncogene rhombotin-2 interacts with retinoblastoma-binding protein 2. Oncogene.

[138] Zhou W, Chen H, Zhang L (2009). The PcG protein hPc2 interacts with the N-terminus of histone demethylase JARID1B and acts as a transcriptional co-repressor. BMB Rep..

[139] Roesch A (2006). Re-expression of the retinoblastoma-binding protein 2-homolog 1 reveals tumor-suppressive functions in highly metastatic melanoma cells. J. Invest. Dermatol..

[140] Tan K (2003). Human PLU-1 Has transcriptional repression properties and interacts with the developmental transcription factors BF-1 and PAX9. J. Biol. Chem..

